# Electroacupuncture versus Moxibustion for Irritable Bowel Syndrome: A Randomized, Parallel-Controlled Trial

**DOI:** 10.1155/2015/361786

**Published:** 2015-07-30

**Authors:** Yin Shi, Yue-Hua Chen, Xiao-Jun Yin, An-Qi Wang, Xing-Kui Chen, Jin-Hua Lu, Rong Ji, Chun-Hui Bao, Jie Sun, Ji-Meng Zhao, Huan-Gan Wu

**Affiliations:** ^1^Department of Medical Clinic, Yueyang Chinese and Western Medicine Integrated Hospital, Shanghai University of Traditional Chinese Medicine, Shanghai 200437, China; ^2^Department of Gastroenterology, Jinhua Municipal Central Hospital, Jinhua 321000, China; ^3^Department of Acupuncture and Moxibustion, Jinhua Municipal Central Hospital, Jinhua 321000, China; ^4^Department of Medical Imaging, Jinhua Municipal Central Hospital, Jinhua 321000, China

## Abstract

*Objective.* To compare the impacts of electroacupuncture (EA) and mild moxibustion (Mox) on patients with irritable bowel syndrome (IBS).* Method.* Eighty-two IBS patients were randomly allocated into EA group (*n* = 41) and Mox group (*n* = 41) and received corresponding interventions for four weeks. Before and after the treatment, the Visual Analogue Scale for Irritable Bowel Syndrome (VAS-IBS) was used to evaluate the gastrointestinal symptoms and mental well-being; and the expression of serotonin (5-hydroxytryptamine, 5-HT), 5-HT_3_ receptor (5-HT_3_R), and 5-HT_4_ receptor (5-HT_4_R) in sigmoid mucosal tissue were detected.* Results*. Both EA and Mox can radically improve the total VAS-IBS score (*P* < 0.05), and EA was found to be more effective in ameliorating the symptom of constipation, while Mox was found to be more effective in ameliorating the symptom of diarrhoea. The abnormal expressions of 5-HT, 5-HT_3_R, and 5-HT_4_R in both groups were significantly improved after the treatments (all *P* < 0.05), and EA was superior to Mox in regulating the abnormally decreased 5-HT_4_R expression in IBS patients with constipation (*P* < 0.05).* Conclusion*. Electroacupuncture and mild moxibustion were both effective in improving IBS symptoms and modulate abnormal expressions of 5-HT, 5-HT_3_R, and 5-HT_4_R in the colonic tissue.

## 1. Introduction

Irritable bowel syndrome (IBS) is a chronic, recurrent functional gastrointestinal (GI) disorder [1] characterized by lower abdominal pain and/or discomfort accompanied by altered defecation without corresponding evidence of structure abnormalities [2]. The general prevalence of IBS around the world was approximately 11% [3] and suggested a female predominant tendency [4]. The first presentation of IBS patient to a physician is mostly between the age of 30 and 50 years, and the symptoms' onset is closely related to psychological stress [5]. Though IBS is unlikely to develop into serious organic disease or has an impact on mortality, it can greatly compromise patients' quality of life and hinder their normal social function [6].

Treatments of IBS range from pharmacological treatments (linaclotide, antispasmodics, 5-HT_3_ antagonists, etc.) [7] to psychological therapies (cognitive behavioural therapy and hypnotherapy) [8]. However, current medications for IBS were targeted on specific dominant symptom [9] while IBS patients always suffer from a group of coexisting complains. Psychological therapies were considered lack of cost efficiency comparing to its moderate severity. Therefore, the most urgent and intractable problem for clinical gastroenterologists to deal with is to develop a both effective and economical treatment method which could cope with different symptoms of IBS.

In recent decades, complementary and alternative medicine, especially Chinese Medicine represented by acupuncture, has been brought into researchers' sights for its substantial therapeutic potency in managing variety of functional disorders and pain syndromes.

So far, a large number of experimental studies have provided robust evidence to support the efficiency of acupuncture/moxibustion in IBS treatment and to illuminate the mechanism underlying their multifunction and multitarget effects [10]. Meta-analysis on randomized sham-controlled trials has verified the therapeutic efficiency of acupuncture in IBS management [11] and further suggested its superiority in achieving GI symptoms amelioration when comparing to conventional pharmacological therapies [12]. Electroacupuncture is a modern adaptation of traditional Chinese hand-manipulated acupuncture. By adding a direct electrical current to needles, the stimulation to the acupoints can be amplified and therefore improve its therapeutic effect. Electroacupuncture was reported to have positive effect on modulating gastrointestinal motility, secretion [13], pain sensation [14], and brain-gut interaction [15]. On the other hand, as another highly efficient Chinese Medicine therapy basing on the Meridians and Acupoints Theory, moxibustion has drawn much less attention than acupuncture, despite its equally robust effectiveness in clinical practice. Existing clinical studies concerning moxibustion therapy on IBS were barely persuasive due to their poor adherence to standard randomized controlled trials (RCTs) criterion and consequently high risk of bias [16].

For this reason, we designed this randomized, parallel-controlled trial strictly following the CONSORT 2010 statement's guideline [17], in order to provide credible clinical evidence to support the application of acupuncture/moxibustion in IBS treatment. Also, we expected that this study might initially prove the distinguishing effects of electroacupuncture and mild moxibustion on different symptoms of IBS and thus can help clinicians to select the optimal treatment based on patients' individual circumstances.

## 2. Materials and Methods

### 2.1. Study Design

This study was a parallel randomized controlled trial approved by the Chinese Clinical Trial Register Centre (registration number: ChiCTR-TRC-11001349). All the participants were randomly allocated into EA group or Mox group by a 1 : 1 ratio.

#### 2.1.1. Participants

All participants were recruited from outpatients of the Department of Gastroenterology in Jinhua Municipal Central Hospital between January 2012 and September 2013.

Eligibility criteria for participants were adults (aged 18–65 years), with symptoms consistent with IBS in accordance with Rome III diagnostic criteria [9], who were willing to participate in the study and sign the informed consent. Patients were to have had symptoms for at least three months and had diarrhea or/and constipation occurring for at least 2 days/week. Other physical diseases including cerebral vessels diseases, liver or kidney diseases, disorders of hematopoietic system, or structural disease on the intestines were required to be absent or inactive. Exclusion criterion also includes formal diagnosis of psychiatric disease, receiving of any medication aiming to treat IBS or that may induce IBS-related symptoms, moxibustion or acupuncture treatment aiming for the treatment of IBS within 2 weeks ahead of the treatment session, and women during pregnancy or nursing.

All the treatment interventions were performed by qualified and experienced acupuncture and moxibustion practitioners from Department of Medical Clinic, Yueyang Chinese and Western Medicine Integrated Hospital affiliated to Shanghai University of Traditional Chinese Medicine, who have the Chinese Medicine practitioner license from the Ministry of Health of China.

#### 2.1.2. Randomization and Blinding

Adopting the SNOSE (sequentially numbered, opaque sealed envelopes) method [18], simple concealed randomization was carried out to eliminating possible selection bias [19]. Participants were allocated into electroacupuncture (EA) group or mild moxibustion (Mox) group on 1 : 1 bases.

To avoid bias and intentional manipulation as far as possible, the initial symptom evaluation, electroacupuncture or mild moxibustion intervention, posttreatment symptom evaluation, and data analysis were performed by different practitioners who were blinded to treatment arm assignments throughout the study. Moreover, the practitioner who performed the electroacupuncture or mild moxibustion treatment was not allowed to exchange any idea with the participants concerning their present symptoms or medical history during the whole treatment session.

This study has been approved by Ethics Committee of Yueyang Hospital of Integrated Traditional Chinese and Western Medicine, Shanghai University of Traditional Chinese Medicine, of the research project (Approved number 2010-08). All patients have been notified of their rights and signed an informed consent.

### 2.2. Interventions

Each patient received a total of 24 EA/Mox treatment sessions, once a day over four weeks, suspended on each Sunday. Acupoints selection and EA/Mox treatment were performed according to Chinese Medicine theory. The acupoints ST-25 (*Tianshu*) and ST-37 (*Shangjuxu*) on both sides of body were located following the national standard of acupoint location GB-12346-90 [20].

For the EA group, after 3 cm radius around the acupoints was sterilized with 75% ethanol, disposable stainless steel needles (0.30 × 40 mm, Hwatuo, Suzhou, China) were inserted into the skin to a depth of 20–25 mm. Twirling-rotating method was then applied to acquire a dull needling sensation called “*De Qi.*” After “*De Qi,*” the electrical leads of the HAN Acupoint Nerve Stimulator (HANS, Model LH 100A TENS, Nanjing, China) were connected to each needle, with stimulation frequency of 2 Hz and intensity of 3.0 mA for 30 min.

The Mox group received mild moxibustion on the same four acupoints for 30 min. Moxa rolls (18 × 200 mm, Hwatuo, Suzhou, China), with one end ignited, were hold vertically to the skin, 2-3 cm above the acupoints. The surface temperature of the acupoints was maintained at 46°C ± 1°C by adapting the distance between the moxa roll and the acupoint. For every 3-4 min, the practitioners would flick the moxa ash into a tray in case the ash falls onto the skin and burns the participant.

### 2.3. Outcome Measures

#### 2.3.1. Primary Outcome Measure

The prespecific primary outcome measure is the total score of the Visual Analogue Scale for Irritable Bowel Syndrome (VAS-IBS) [21, 22]. The VAS-IBS was a self-rating questionnaire, developed basing on the widely used Visual Analogue Scale (VAS), to measure treatment response of gastrointestinal symptoms as well as mental well-being in patients with IBS. As exhibited in Table 1, VAS-IBS questionnaire contains seven items covering five most common gastrointestinal symptoms (abdominal pain, diarrhoea, constipation, bloating and flatulence, and vomiting and nausea), mental well-being, and the impact of IBS symptoms on daily life. The score for each item ranges from 0 (most severe) to 100 (absent of symptom), resulting in a total score between 0 and 700. This questionnaire was translated and modified into Chinese in line with practical demand.

#### 2.3.2. Secondary Outcome Measure

The secondary outcome measures are the five gastrointestinal symptoms from the VAS-IBS. The improvement or aggravation of each single symptom was demonstrated by the differential of scores before and after the interventions. By comprising these differentials, the therapeutic superiority of EA and Mox treatments on different gastrointestinal symptoms was able to be evaluated.

#### 2.3.3. Immunohistochemistry

Before and after the treatment, all participants received colonoscopy with or without intravenous anaesthesia, and sigmoid colon tissues were taken to detect the expression of 5-HT, 5-HT_3_R, and 5-HT_4_R. At the same time, 10 sigmoid colon tissue samples from healthy volunteers were taken as normal control (NC). All samples were first fixed in 10% neutral-buffered formalin, and then tissues vertical to intestinal diameter were selected, dehydrated, and embedded in paraffin. After being sliced into 4 *μ*-thick paraffin sections and baked at 58°C for 24 h, the expression of 5-HT, 5-HT_3_R, and 5-HT_4_R in colon tissue was detected by immunohistochemical staining. The sections were exposed to 0.01 M CB, pH 6.0, microwaved at 30% power for 20 min for heat fixation, and cooled to room temperature. The sections were washed 3 times with PBS for 3 min and exposed to 0.3% H_2_O_2_ for 20 min at room temperature to inhibit endogenous peroxidases. Following a final PBS wash (3 × 3 min), the samples were exposed to 20% normal goat serum and incubated for 30 min. Antibodies were added dropwise (5-HT 1 : 100, 5-HT_3_R 1 : 50, and 5-HT_4_R 1 : 80, Santa Cruz, CA), and the sections were incubated at 37°C for 2 hours. The sections were washed with PBS 3 times for 3 min, incubated in HRP/R reagent at 37°C for 30 min, and PBS washed 3 times for 3 min. The sections were then incubated in DAB chromogenic reagent for 8 to 12 min and dyed with hematoxylin lining and blue in the presence of hot water. After drying, the sections were wrapped with neutral gum. A semiquantitative analysis of the staining was performed using the MIQAS medical image quantitative analysis system (Shanghai Qiuwei Biomedical Technology Company). Positive results were indicated by the presence of brown or tan particles in the stained colonic tissue cells. In each slice, three positive areas were counted and assessed for optical density (OD) in a high power field to calculate an immunohistochemical positive index (IHC index = positive area × OD/total area) for 5-HT, 5-HT_3_R, and 5-HT_4_R.

### 2.4. Statistical Methods

Statistical analyses were performed using SPSS 18.0 (SPSS Inc., Chicago, Illinois). Normally distributed continuous variables were expressed as mean ± SD, while abnormally distributed continuous variables were expressed as mean, the 25th, and the 75th percentiles. Categorical variables were showed as frequencies and proportions. For normally distributed continuous variables, differences within groups before and after the treatment sessions were compared by paired-samples *t*-test, and differences between groups were compared by two independent samples *t*-test. Abnormally distributed continuous variables were compared by Wilcoxon rank sum tests. Pearson's *χ* ^2^ test was used for comparisons of categorical variables. Differences were considered statistically significant if *P* < 0.05.

## 3. Results

### 3.1. Participant Flow

**Figure 1:**
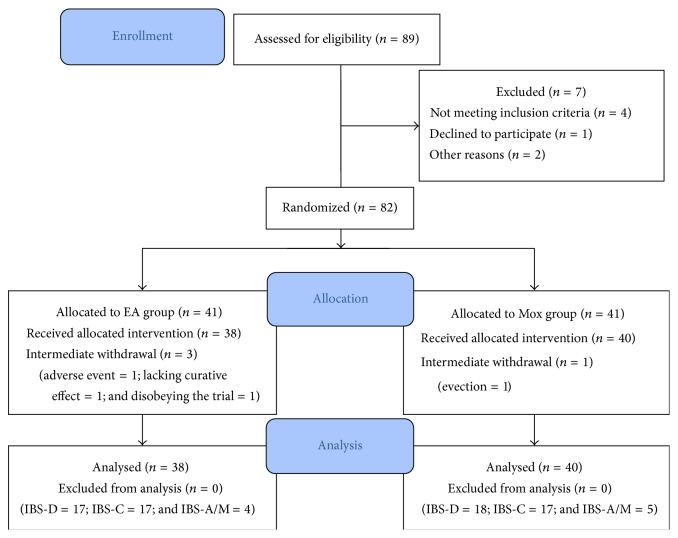
Flow diagram of the study. Flow gram for the trial. EA: electroacupuncture, Mox: mild moxibustion.

Figure 1 is a diagram illustrating the participant flow of the study. From January 2012 to September 2013, 89 participants were recruited, of which 7 were excluded, leaving 82 who were randomly allocated into EA group or Mox group. The majority of eligible participants (*n* = 82, 92.13%) completed the trials, while 4 (4.88%) withdrawn the treatments midway.

Scores of the VAS-IBS questionnaire were measured immediately prior to the first treatment session, and again at the point the last treatment session was finished. In the end, data from 78 participants were included for the final analysis.

### 3.2. Baseline Data

Baseline demographic data include gender and age, disease duration, previous medication, VAS-IBS total score, and IBS subtype distribution in each group (Table 2). No significant differences were demonstrated in these parameters between the two treatment groups (*P* < 0.01).

### 3.3. Outcomes and Estimation

#### 3.3.1. Primary Outcome Measure

The participants' overall perception of their gastrointestinal symptoms and their subjective mental well-being was translated into quantitative parameters by the VAS-IBS total score. Within-group and between-group comparison were made to evaluate treatment responses to each intervention method. A remarkable decline in VAS-IBS total scores was identified after the whole treatment sessions in both EA group and Mox group (*P* < 0.01). The improvement of VAS-IBS total score in Mox group was slightly greater than that of EA group; however, between-group difference showed no statistical significance (Table 3).

#### 3.3.2. Secondary Outcome Measure

**Figure 2:**
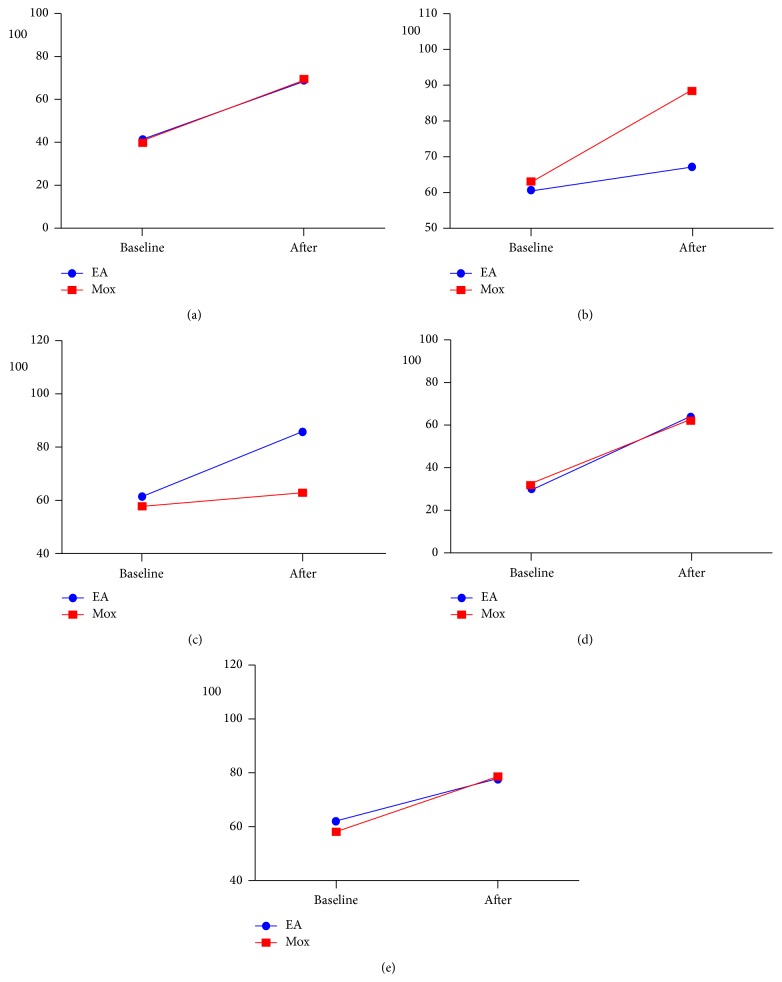
Secondary outcome measure of the VAS-IBS score. Secondary outcome changes in VAS-IBS gastrointestinal symptom scores at baseline and after treatment. (a) Abdominal pain, (b) diarrhoea, (c) constipation, (d) bloating and flatulence, and (e) vomiting and nausea. EA: electroacupuncture, Mox: mild moxibustion.

The five items from the VAS-IBS questionnaire concerning gastrointestinal symptoms, namely, abdominal pain, diarrhoea, constipation, bloating and flatulence, and vomiting and nausea, were utilized as secondary outcome measures to assess the impact of EA and Mox intervention on each specific symptoms. In EA group, all the five symptoms were significantly improved after the treatment (*P* < 0.01). Between-group comparisons of the change in score of each symptom were illustrated in Figure 2. Differences in therapeutic effect of EA and Mox interventions on the symptoms of abdominal pain, bloating and flatulence, and vomiting and nausea were barely noticeable. However, EA demonstrated a significant grater advantage in ameliorating the symptom of constipation in IBS participants (24.29 versus 5.27, *P* < 0.01). Conversely, Mox was significantly more efficient in ameliorating the symptom of diarrhoea (25.42 versus 6.55, *P* < 0.01).

#### 3.3.3. 5-HT Expressions before and after the Treatment

**Figure 3:**
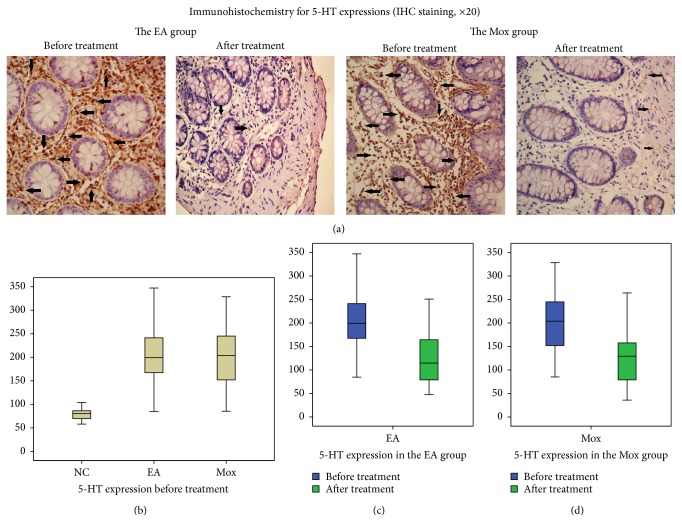
Comparison of 5-HT expressions before and after treatment. (a) Immunohistochemistry for 5-HT expressions in sigmoid mucosa tissue of IBS patients before and after treatments in the EA and Mox groups (IHC staining, ×20). Arrows represent immunohistochemistry positive expressions. (b) 5-HT expressions in the EA and Mox groups before treatment comparing to the NC group, (c) 5-HT expression before and after the treatment in the EA group (*P* < 0.01), and (d) 5-HT expression before and after the treatment in the Mox group (*P* < 0.01). NC: the normal control group, EA: the electroacupuncture group, and Mox: the mild moxibustion group.

Figure 3(a) showed the immunohistochemical positive area of 5-HT expressions in IBS patients' sigmoid colon tissues before and after the EA and Mox treatments. Before the treatment (Figure 3(b)), the 5-HT expressions in the colonic tissue of the EA and Mox groups were both significantly higher than that of the NC group (204.37 ± 61.97 and 198.93 ± 65.30 versus 78.67 ± 13.88, resp., both *P* < 0.01), while no significant difference was observed between the EA and Mox groups (*P* = 0.905, *P* > 0.05).

After the treatment (Figures 3(c) and 3(d)), the abnormally increased expressions of 5-HT in the colonic tissue of the EA and Mox groups were both significantly decreased (122.13 ± 50.09 versus 204.37 ± 61.97 and 123.21 ± 50.06 versus 198.93 ± 65.30, resp., both *P* < 0.01). However, by comparing the differentials in 5-HT expressions before and after the treatments, no significant difference was observed between the EA and Mox groups (−82.23 ± 33.57 versus −75.72 ± 39.97, *P* = 0.190, *P* > 0.05).

#### 3.3.4. 5-HT_3_R Expressions before and after the Treatment

**Figure 4:**
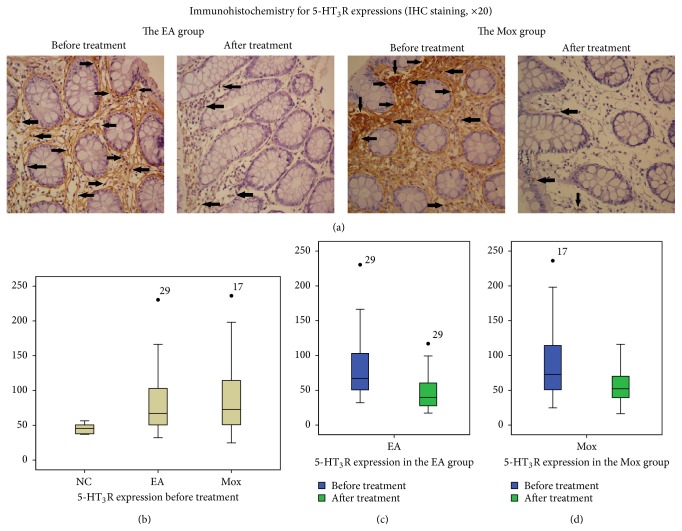
Comparison of 5-HT_3_R expressions before and after treatment. (a) Immunohistochemistry for 5-HT_3_R expressions in sigmoid mucosa tissue of IBS patients before and after treatments in the EA and Mox groups (IHC staining, ×20). Arrows represent immunohistochemistry positive expressions. (b) 5-HT_3_R expressions in the EA and Mox groups before treatment comparing to the NC group, (c) 5-HT_3_R expression before and after the treatment in the EA group (*P* < 0.01), and (d) 5-HT_3_R expression before and after the treatment in the Mox group (*P* < 0.01). NC: the normal control group, EA: the electroacupuncture group, and Mox: the mild moxibustion group.

Figure 4(a) showed the immunohistochemical positive area of 5-HT_3_R expressions in IBS patients' sigmoid colon tissues before and after the EA and Mox treatments. Before the treatment (Figure 4(b)), the 5-HT_3_R expressions in the colonic tissue in the EA and Mox groups were both significantly higher than the NC group (79.75 ± 42.72 and 90.08 ± 50.70 versus 45.19 ± 6.84, both *P* < 0.01), while no significant difference was observed between the EA and Mox group (79.75 ± 42.72 versus 90.08 ± 50.70, *P* = 0.472, *P* > 0.05).

After the treatment (Figures 4(c) and 4(d)), the abnormally increased 5-HT_3_R expressions in the colonic tissue of the EA and Mox groups were both significantly decreased (66.98 (50.01, 103.96) versus 39.63 (27.40, 61.19), and 72.73 (49.41, 115.09) versus 52.23 (39.18, 70.60), resp., both *P* < 0.01). However, by comparing the differentials in 5-HT_3_R expressions before and after the treatments, no significant difference was observed between the EA and Mox groups (*P* = 0.335, *P* > 0.05).

#### 3.3.5. 5-HT_4_R Expressions before and after the Treatment

**Figure 5:**
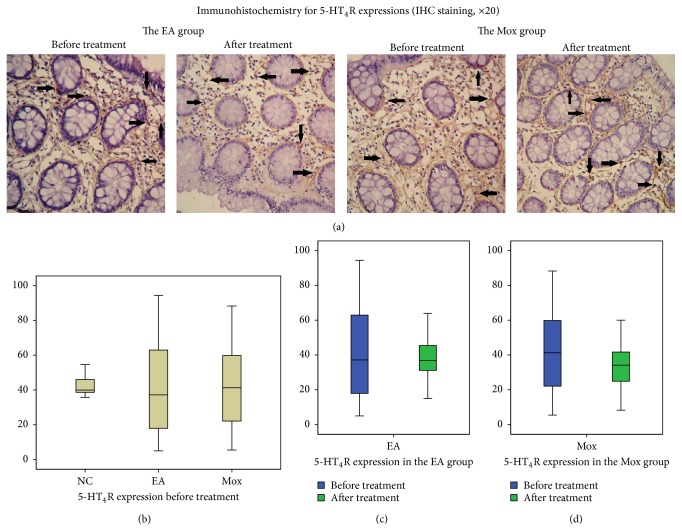
Comparison of 5-HT_4_R expressions before and after treatment. (a) Immunohistochemistry for 5-HT_4_R expressions in sigmoid mucosa tissue of IBS patients before and after treatments in the EA and Mox groups (IHC staining, ×20). Arrows represent immunohistochemistry positive expressions. (b) 5-HT_4_R expressions in the EA and Mox groups before treatment comparing to the NC group, (c) 5-HT_4_R expression before and after the treatment in the EA group, and (d) 5-HT_4_R expression before and after the treatment in the Mox group (*P* < 0.01). NC: the normal control group, EA: the electroacupuncture group, and Mox: the mild moxibustion group.

Figure 5(a) showed the immunohistochemical positive area of 5-HT_4_R expressions in IBS patients' sigmoid colon tissues before and after the EA and Mox treatments. Before the treatment (Figure 5(b)), between-group comparison among the EA, Mox and NC groups showed no significant differences in 5-HT_4_R expressions in the colonic tissue (39.97 (38.03, 46.87) and 37.18 (17.93, 64.01) versus 41.27 (21.89, 59.87), all *P* > 0.05). However, the tendency of dispersion in the EA and Mox groups was much greater than that of the NC group.

After the treatment (Figures 5(c) and 5(d)), there were no significant differences in the expression of 5-HT_4_R in the EA group before and after the treatment (37.18 (17.93, 64.01) versus 36.83 (31.05, 45.57), *P* = 0.446, *P* > 0.05), while the expression of 5-HT_4_R in the Mox group was significantly decreased after the treatment (41.27 (21.89, 59.87) versus 34.14 (24.27, 42.11), *P* = 0.002, *P* < 0.01). By comparing the differentials in 5-HT_4_R expressions before and after the treatments, no significant difference was observed between the EA and Mox groups (*P* = 0.079, *P* > 0.05). However, the absolute values of these differentials showed that changes in 5-HT_4_R expressions of the EA group were significantly greater than that of the Mox group (15.21 (7.57, 20.20) versus 5.89 (2.77, 16.45), *P* = 0.015, *P* < 0.05).

#### 3.3.6. Subgroup Analysis of Different IBS Subtypes' Differentials in 5-HT_4_R Expression before and after the Treatment

Table 4 demonstrated 5-HT_4_R expressions in the colonic tissue of each IBS subtype before and after the treatments in the EA and Mox groups. Before the treatments, 5-HT_4_R expressions in each IBS subtype showed no significant difference in the EA and Mox group (*P* = 0.574, 0.189, 0.610 for IBS-D, IBS-C, and IBS-A/M, resp., all *P* > 0.05).

In IBS-D patients, 5-HT_4_R expressions in the colonic tissue of the EA and Mox groups were both significantly higher than that of the NC group (65.56 ± 15.82 and 62.88 ± 12.00 versus 42.07 ± 6.04, both *P* < 0.01). After the treatments, these abnormally increased 5-HT_4_R expressions were both significantly decreased (both *P* < 0.01 versus before the treatment). However, by comparing the differentials in 5-HT_4_R expressions of IBS-D patients before and after the treatments, no significant difference was observed between the EA and Mox groups (*P* = 0.385, *P* > 0.05).

In IBS-C patients, 5-HT_4_R expressions in the colonic tissue of the EA and Mox groups were both significantly lower than that of the NC group (18.37 ± 7.83 and 22.42 ± 9.69 versus 42.07 ± 6.04, both *P* < 0.01). After the treatments, these abnormally increased 5-HT_4_R expressions were both significantly decreased (both *P* < 0.01 versus before the treatment). At the same time, the increase of 5-HT_4_R expression in the EA group was found to be significantly greater than that of the Mox group (*P* < 0.01).

In IBS-A/M patients, between-group comparison showed no significant differences in 5-HT_4_R expressions in the colonic tissue between any two of the three groups (37.07 ± 8.19 versus 32.62 ± 14.81 versus 42.07 ± 6.04, all *P* > 0.05). After the treatments, within-group comparison showed no significant difference in the 5-HT_4_R expression of the EA group (*P* = 0.456 versus before the treatment) and a significant decrease in the 5-HT_4_R expression of the EA group (*P* = 0.015 versus before the treatment, *P* < 0.05). However, by comparing the differentials in 5-HT_4_R expressions of IBS-A/M patients before and after the treatments, no significant difference was observed between the EA and Mox groups (*P* = 0.624, *P* > 0.05).

### 3.4. Treatment Adherence

Thirty-eight out of 41 participants (92.68%) in the EA group completed the trial, in comparison to 40 out of 42 (97.56%) in the Mox group. Among the three participants who failed to complete the treatment sessions in the EA group, one disobeyed the trial for taking medication aiming for the treatment of IBS; one lacked curative effect and asked to withdraw voluntarily; only one was afraid of acupuncture and refused to continue the trial during the first treatment session. The one withdrawn participant in Mox group was due to long time evection.

### 3.5. Safety Control and Adverse Events

To minimize the occurrence rate of adverse events, a care provider was specially assigned to explain the dos and don'ts regarding EA and Mox treatments before the treatment started. Emphasis has been drawn on avoiding the state of hunger, fatigue, and drunkenness. Warm water and candy bars were prepared in the case that participants developed a symptom of acupuncture fainting. All the treatments were applied after the participant was lying on the bed in a comfortable position.

Two participants in the EA group (5.26%) and two in the Mox group (5%) reported discomfort such as mild sickness, sweating, or dizziness during the treatment sessions. Most incidents occurred at the first session and were relieved after resting. All these four participants continued and finished the trial.

## 4. Discussion

### 4.1. Acupuncture and Moxibustion in IBS Management

IBS is a functional disorder whose leading symptom is diarrhoea or constipation-associated abdominal pain or discomfort. By the nature of altered bowel habits, IBS is classified into subtypes including IBS-C, irritable bowel syndrome with constipation; IBS-D, irritable bowel syndrome with diarrhoea; IBS-M, mixed irritable bowel syndrome (with both diarrhea and constipation >25% of bowel movements); and IBS-A, alternating irritable bowel syndrome (bowel habits often vary over time) [1]. This classification was originally designed to help selecting patients for treatments or clinical trials targeting a specific bowel pattern. However, this single symptom oriented strategy was largely disabled by the rapidly fluctuating symptoms in IBS patients [23]. By contrast, this unpredictable characteristic of symptoms is barely problematic in acupuncture and/or moxibustion practice because of these two therapies focusing on the principle cause of a disease rather than some specific symptoms. The result showed that although the symptoms of participants were varied and their IBS subtypes were different, most of them were well responded to their allocated treatment. What needs to be noticed is that this symptom amelioration made be EA/Mox was not confined to one or some specific symptoms, but rather universal. At the point that the treatment session was ended, a majority of participant reported that most of their IBS-related symptoms were obviously relived, but the improvement of each symptom was not simultaneous. In most case, abdominal pain and distension sensation were the first improved symptoms simply after one or two sessions. By contrast, the improvement of diarrhoea or/and constipation, measured by defecation frequency and stool form, was rather a gradual procedure.

The most intriguing finding in this study is the different advantage of EA/Mox therapy in IBS treatment. Though their efficiency in improving the VAS-IBS score had no significant difference, EA appeared to be more potent when dealing with constipation, while Mox demonstrated the advantage in coping diarrhoea.

In recent years, researchers have gained concrete evidence to support the efficiency of these ancient therapies and were making constant endeavour to illuminate their functioning mechanisms including affecting visceral sensation, motility, and/or brain-gut interactions [24–26]. At the same time, the different mechanisms of these two therapies were also identified by a large amount of researches.

Electroacupuncture (EA) is a combination of electrical impulse and acupuncture marked by its outstanding efficacy in alleviating both sensory and affective inflammatory pain [27], including visceral neuropathic pain in IBS, and improving visceral hyperalgesia [28] toward visceral stimuli. At the same time, it can also promote gastrointestinal motility [29] in patients with constipation. In the current research, we actually observed the superiority of EA in alleviating the symptom of constipation after treatment comparing to Mox. Those participants who originally complained about prolonged defecation, straining, and/or feeling of incomplete evacuation reported a notable improvement in bowel habits and stool form. Some participants from the EA group reported subjective feeling of accelerated gastrointestinal peristalsis and increased appetite during the treatment sessions, also consequently weight gain.

As to moxibustion, former study showed that the thermal stimulation of moxibustion to the paraumbilical region can increase blood flow of superior mesenteric artery [30] and relieve abdominal pain due to vasospasm. Nevertheless, recent study on mice suggested the potential of moxibustion in antibacterial infection by activating macrophage autophagy [31]. In this study, comparing between Mox and EA has demonstrated the significant superiority of Mox in improving diarrhoea in IBS, especially in participants whose diarrhoea often occurs after the intake of cold drink/food, or when exposed in cold environments. This kind of participants was usually more sensitive to Mox therapy because their hypersensitivity to cold stimuli could be principally improved by the warm stimulation of Mox.

### 4.2. Serotonin and Its Receptors in Pathophysiology and Treatment of IBS

The interaction between the central nervous system (CNS) and enteric nervous system (ENS) through various neurotransmitters and hormones composes a complex bidirectional signalling system called brain-gut axis [32]. 5-HT, as one of the brain-gut peptides locating in both CNS and ENS, plays a predominant role in the pathophysiology of IBS [33]. Through interaction with different receptors, 5-HT controls the intestinal motility and secretion. Abnormalities in 5-HT signalling system may affect the sensory, motor, and secretory function of the digestive system, result in gastrointestinal dysmotility, visceral hypersensitivity, and infection, and further influence patients' mental condition [34].

The signal transmission between the CNS and ENS is predominantly mediated by 5-HT_3_R [35], and the significantly lowered pain threshold and hyperactivity of the gastrointestinal smooth muscles in IBS patients are closely related to the increased 5-HT_3_R in gastrointestinal tract [36]. Previous studies have showed that both EA and Mox were efficient in enhancing the pain threshold and downregulating the abnormally increased 5-HT and 5HT_3_R expressions, therefore relieving hypersensitivity [37–39]. The current research found that, before treatment, IBS patients exhibited an elevated expression of 5-HT and 5-HT_3_R in colon tissue compared to healthy controls. Following EA and Mox treatment, the expression of 5-HT and 5-HT_3_R in colon tissue was significantly decreased along with the significant alleviation of abdominal pain, bloating, or abdominal discomfort. These results confirmed that overexpression of 5-HT and 5-HT_3_R expressions in the colon tissue of IBS patients is closely related to visceral hypersensitivity and further suggested that both EA and Mox treatments may relieve symptoms result from visceral hypersensitivity by downregulating of 5-HT and 5-HT_3_R expressions.

5-HT_4_R is another important receptor in regulating gastrointestinal function. By acting at 5-HT_4_R located in gastrointestinal mucosa and smooth muscles, 5-HT is able to release transmitters in prokinetic reflex pathways and therefore promote and maintain propulsive intestinal motility [40]. In clinical practice, 5-HT_4_R agonists are widely used in the management of IBS-C to promote gastrointestinal motility and attenuate visceral pain [41]. The current research found that, comparing to the healthy controls, 5-HT_4_R expression in the colonic mucosa was significantly lower in IBS-C patients and significantly higher in IBS-D patients, suggesting that the abnormal expression of 5-HT_4_R is involved in gastrointestinal motility disorder. These findings suggested that EA therapy can significantly promote intestinal peristalsis of IBS-C patients and effectively alleviate symptoms including defecation frequency, difficulty in defecation, and constipation, and it might work through increasing 5-HT_4_R expression in the colonic mucosa. Conversely, moxibustion therapy had no apparent effect on 5-HT_4_R expression in the colonic mucosa of C-IBS patients, and the defecation frequency and constipation were not improved in these patients.

### 4.3. Strength and Limitation

Currently, many studies have been devoted to prove the clinical feasibility and efficiency of acupuncture/moxibustion in treating IBS. However, despite the encouraging evidence from animal experiment, most findings from these clinical studies were much less satisfactory and persuasive largely because of their questionable randomization process and poorly standardized intervention procedure [12]. In this study, we have designed and conducted the study according to the standard RCT principle in CONSORT 2010 statement [17] and CONSORT extension for acupuncture. During the whole procedure, we make a great deal of efforts to make sure the randomization and blinding were obeyed, although double-blind trial is very difficult to achieve in trials involving acupuncture/moxibustion intervention because practitioners need to ask the patient's feeling to make sure the acquisition of* De Qi* sensation, and this specific sensation is closely related to the therapeutic effect of these therapies. We hope that our findings here would provide the initial reliable scientific evidence for the clinical utility of acupuncture/moxibustion in IBS management. Also, the different finding in EA and Mox treatment might help the acupuncture/moxibustion clinicians to select a more appropriate therapy for each IBS individual basing on their diarrhoea/constipation symptoms.

## Figures and Tables

**Table 1:** VAS-IBS questionnaire.

Items	Score range
Gastrointestinal symptoms	
Abdominal pain	0–100
Diarrhoea	0–100
Constipation	0–100
Bloating and flatulence	0–100
Vomiting and nausea	0–100
Mental health	
Perception of mental well-being	0–100
Quality of life	
GI symptom influencing daily life	0–100

Total score	0–700

0 represents very severe problems and 100 represents absence of problems. VAS-IBS: Visual Analogue Scale for Irritable Bowel Syndrome, GI: gastrointestinal.

**Table 2:** Baseline demographic characteristics and VAS-IBS total scores.

Parameter	Group EA (*n* = 41)	Group Mox (*n* = 41)
Gender		
Female (%)	60.98	65.29
Male (%)	39.02	34.71
Age yr (mean, (min, max))	39.45 (19, 61)	40.26 (20, 64)
Course of disease yr (mean ± SD)	6.47 ± 3.84	7.28 ± 5.44
Current medication		
Yes (%)	0 (0)	0 (0)
No (%)	41 (100.00)	41 (100.00)
VAS-IBS total score (mean ± SD)	253.49 ± 45.93	250.24 ± 41.08
IBS subtype		
IBS-D (%)	17 (41.46)	18 (43.90)
IBS-C (%)	19 (46.34)	17 (41.46)
IBS-A/IBS-M (%)	5 (12.20)	6 (14.63)

Visual Analogue Scale for Irritable Bowel Syndrome; EA: electroacupuncture; Mox: moxibustion; SD: standard deviation; IBS-C: irritable bowel syndrome with constipation; IBS-D: irritable bowel syndrome with diarrhoea; IBS-M: mixed irritable bowel syndrome (with both diarrhoea and constipation >25% of bowel movements); and IBS-A: alternating irritable bowel syndrome (bowel habits often vary over time).

**Table 3:** IBS-VAS total score before and after the treatment sessions.

Group	*n*	Before treatment (mean ± SD)	After treatment (mean ± SD)	Differentials
EA	38	253.66 ± 46.02	363.61 ± 75.71^▲^	109.95
Mox	40	250.17 ± 41.19	362.00 ± 65.14^▲^	111.83

^▲^ *P* < 0.01 versus before the treatment. Visual Analogue Scale for Irritable Bowel Syndrome; EA: electroacupuncture; Mox: moxibustion; and SD: standard deviation.

**Table 4:** Subgroup analysis of different IBS subtypes' differentials in 5-HT4R expression before and after treatment (mean ± SD).

Group	IBS subtypes	*n*	Before treatment	After treatment	Differentials
EA	IBS-D	17	65.56 ± 15.82	47.03 ± 10.21^▲^	−18.53 ± 7.77
	IBS-C	17	18.37 ± 7.83	32.39 ± 8.78^▲^	14.03 ± 6.41^*∗*^
	IBS-A/M	4	37.07 ± 8.19	35.03 ± 7.68	−2.04 ± 4.78

Mox	IBS-D	18	62.88 ± 12.00	41.99 ± 8.70^▲^	−20.88 ± 8.02
	IBS-C	17	22.42 ± 9.69	25.57 ± 10.31^▲^	3.14 ± 1.97
	IBS-A/M	5	32.62 ± 14.81	28.67 ± 13.23^△^	−3.94 ± 2.13

^▲^ *P* < 0.01 versus before the treatment; ^△^ *P* = 0.015 versus before the treatment, *P* < 0.05; ^*∗*^ *P* < 0.01 versus the Mox group. EA: electroacupuncture; Mox: moxibustion; SD: standard deviation; IBS-C: irritable bowel syndrome with constipation; IBS-D: irritable bowel syndrome with diarrhoea; IBS-M: mixed irritable bowel syndrome (with both diarrhoea and constipation >25% of bowel movements); and IBS-A: alternating irritable bowel syndrome (bowel habits often vary over time).
